# Comparative analysis of the diversity within the *B. bronchiseptica fimX* locus

**DOI:** 10.1371/journal.pone.0342754

**Published:** 2026-03-02

**Authors:** Tracy L. Nicholson, Sarah M. Shore

**Affiliations:** National Animal Disease Center, Agricultural Research Service, USDA, Ames, Iowa, United States of America; RV University, INDIA

## Abstract

*Bordetella bronchiseptica* is a highly contagious veterinary bacterial respiratory pathogen with a broad host range that can cause a variety of clinical disease outcomes ranging from asymptomatic carriage to severe pneumonia. *B. bronchiseptica* fimbriae mediate attachment to respiratory epithelium and are considered to serve as potential protective antigens. Several comparative genomic studies involving *B. bronchiseptica* strains have demonstrated an overall low level of genomic variability. The region located within the *fimX* locus is one of the few regions in which genomic variability has been reported. The goal of this study was to comprehensively evaluate the genomic variability harbored within the *fimX* locus among *B. bronchiseptica* strains. Our analysis revealed that the genetic variability identified within the *fimX* locus included both the number of genes harbored, as well as the type of predicted fimbrial subunit gene types contained within the *fimX* locus. A total of six different fimbrial subunit gene types were identified among the *B. bronchiseptica* genome assemblies analyzed in this study. Comparative analysis data also suggests that the diversity among the fimbrial subunit genes located within the *fimX* locus was likely generated by homologous recombination.

## Introduction

*Bordetella bronchiseptica* is a highly contagious respiratory bacterial veterinary pathogen. It can serve as the primary cause or as a contributor to a spectrum of clinical disease outcomes ranging from asymptomatic carriage to severe bronchopneumonia [1–3]. *B. bronchiseptica* is closely related to *Bordetella pertussis* and *Bordetella parapertussis*_hu_, the bacterial pathogens responsible for causing whooping cough in humans [4–7]. The human-restricted pathogens, *B. pertussis* and *B. parapertussis*_*hu*_, are regarded to have evolved independently from a *B. bronchiseptica* common ancestor [5–7]. In addition to their close genetic relatedness, *B. bronchiseptica*, *B. pertussis*, and *B. parapertussis*_*hu*_, commonly referred to as the ‘classical bordetellae’, all harbor many of the same virulence factors that are similarly regulated [5,8–10]. Despite these similarities, these *Bordetella* species differ in traits such as host specificity, disease severity, and duration of infection. The host-specific *B. pertussis* infects humans, has no animal reservoir, and lacks the ability to survive in the environment. In contrast, *B. bronchiseptica*, infects a variety of animals, often establishing chronic infections that range from lethal pneumonia to asymptomatic carriage and is capable of surviving in the environment [2,4,11,12]. Although rare and predominately occurring in immunocompromised individuals, human infections of *B. bronchiseptica* have been reported [13–16].

Fimbriae are some of the virulence factors produced by classical bordetellae [1,2,5,8,9]. Vaccines and therapies targeting the fimbrial antigens have been successful in both human and veterinary applications [17–23]. Fimbriae produced by *Bordetella* are considered members of the type I pili family and are assembled and exported by the chaperone–usher pathway, which has been extensively investigated in uropathogenic *Escherichia coli* (UPEC) [24–30]. Studies have demonstrated that *B. bronchiseptica* genomes contain several fimbrial subunit genes, most of which are unlinked and located throughout the chromosome [5,8,9,31–33]. While these major fimbrial subunit genes are unlinked on the chromosome, assembly and export of the fimbrial subunit products are mediated by the products of three genes, *fimB, fimC,* and *fimD* [30,34,35]. FimB shares similarity with the UPED chaperone protein, PapD, thought to prevent degradation of the fimbrial subunit proteins in the periplasmic space. FimC shares similarity with the UPEC usher protein, PapC, considered to aid in the transport of fimbrial subunit proteins across the outer membrane and secure the fimbrial structure [34]. FimD is considered to function as the adhesive tip of the fimbrial structure [36]. Current knowledge regarding the involvement of *B. bronchiseptica* fimbriae in adherence to the respiratory epithelium, as well as the involvement of *B. bronchiseptica* fimbriae as protective antigens, is derived from studies employing a *B. bronchiseptica* strain that is incapable of producing any fimbrial serotypes due to an in-frame deletion of the *fimBCD* genes [35,54]. These studies additionally demonstrate that the *fimBCD* genes function as the sole chaperone-usher system required for the assembly and export of *B. bronchiseptica* fimbrial subunit products [35,54].

Several comparative genomic studies involving *B. bronchiseptica* strains have demonstrated an overall low level of limited genomic variability [5,8,9,31–33]. One of the few regions in which genomic variability has been reported is the region located within the *fimX* locus [5,8,9,31–33]. Given a downstream potential use of fimbrial subunits as vaccine targets, and the high genetic diversity observed, the goal of this report was to comprehensively evaluate the genetic variation and phylogenic relationship of the *fimX* locus among *B. bronchiseptica* strains.

## Materials and methods

### Bacterial strains

*B. bronchiseptica* (taxon ID = 518) Reference Sequence (RefSeq) genome assemblies were obtained from the National Center for Biotechnology Information (NCBI) RefSeq database (https://www.ncbi.nlm.nih.gov/genbank/, accessed in October 2024). Genome assemblies for isolates MBORD624 and 00-P-2796 were subsequently excluded from our dataset due to poor quality. Genome assemblies from *B. bronchiseptica* multi-isolate studies were additionally included in the dataset [31,32]. Multi-locus sequence typing (MLST) was performed *in silico* utilizing the BIGSdb-Pasteur databases hosted by the Institut Pasteur (https://bigsdb.pasteur.fr). In total, 259 *B. bronchiseptica* genome assemblies were included in the dataset. All strains and strain-associated metadata are provided in S1 Table.

### Analysis and categorization of *fimX* loci into cluster types

**Fig 1:**
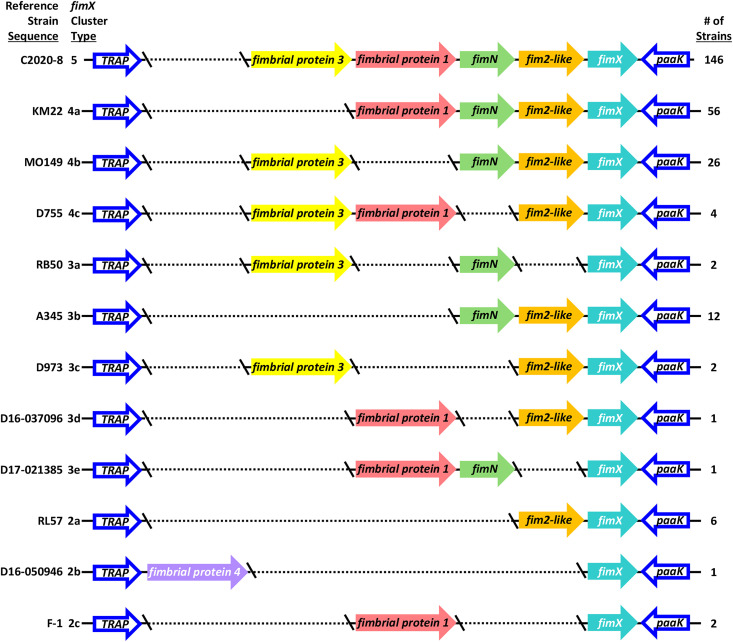
Genomic organization of *fimX* locus among *B. bronchiseptica* genome assemblies. Reference strain sequence and cluster type provided at left. Predicted fimbrial subunit genes are represented as arrows and color-coded by fimbrial subunit type with gene names, defined by nucleotide sequence identity. Dashes indicate break in the sequence.

Genes flanking the *fimX* locus, tripartite ATP-independent periplasmic transporter (*TRAP*) and phenylacetate-CoA ligase (*paaK*), were used to locate and extract the *fimX* locus nucleotide sequence from all NCBI Prokaryotic Genome Annotation Pipeline (PGAP) (versions 6.5–6.8) [37] annotated assemblies. When the *fimX* locus was not located within a single contiguous region, the partial regions were identified by proximity to one of the flanking genes and the presence of one or more fimbrial subunit genes. Nucleotide sequences containing these partial regions were examined to ensure that they did not contain the fimbrial subunit genes *fim2*, *fim3*, *fimA*, or *fimbrial protein 2* (homolog to KM22 CJ015_08855), which are located in other chromosomal locations. The *fimX* loci were categorized into cluster types based on [1] the number of fimbrial subunit genes located within the *fimX* locus and [2] the fimbrial subunit gene types contained within the *fimX* locus. A reference strain sequence was chosen to represent each *fimX* cluster type with preference given to strains with closed whole-genome assemblies. For example, strain C2020-8 was chosen as reference strain for cluster type 5. When no closed whole-genome assemblies were available, preference was given to strains D16-037096 (cluster type 3d), D17-021385 (cluster type 3e), D16-050946 (cluster type 2b), and F-1 (cluster type 2c) because they are maintained in our laboratory isolate collection, which allowed for confirmatory Sanger sequencing and further evaluation. All twelve reference strains and their corresponding cluster types are provided in Fig 1 and S1 Table.

### Sequencing and analysis of *fimX* locus

The *fimX* locus region of strains D16-037096 (Cluster Type 3d), D17-021385 (Cluster Type 3e), D16-050946 (Cluster Type 2b), and F-1 (Cluster Type 2c) was amplified by PCR, sequenced, and further analyzed. Isolate F-1 was obtained from the USDA-ARS Culture Collection (NRRL) and isolates D16-037096, D17-021385, and D16-050946 have been previously described [31]. Strains were grown from frozen stocks (−80°C in 30% glycerol) on Bordet-Gengou agar (Difco, Sparks, MD) supplemented with 10% sheep’s blood and 40 µg/ml Streptomycin.

A single colony was inoculated into Stainer-Scholte broth containing 40 µg/ml Streptomycin and grown overnight at 37°C with shaking. Genomic DNA was isolated using the High Pure PCR Template Preparation Kit (Roche Diagnostics, Mannheim, Germany). The entire fimX locus was PCR amplified using primers TRAP_for and paaK_rev in a PCR reaction with Platinum SuperFi II PCR Master Mix (Invitrogen, Carlsbad, CA) as specified by the manufacturer. PCR products were purified using the Monarch Spin DNA Gel Extraction Kit (New England Biolabs, Ipswich, MA). Sanger sequencing was then performed on purified PCR products with appropriate primers (Table 1). Isolates evaluated were obtained from samples collected as part of previous studies and did not require Institutional Animal Care and Use Committee (IACUC) approval.

**Table 1:** Primers used in study.

Primer Name	Sequence (5’ to 3’)
TRAP_for	ATGTCGCAGTTGATGATTCTCTCGA
paaK_rev	ATGTCCACCACCATGAGCAAGCCGG
fp3-for	TCGCATCCTTAGTGACATAG
fimN-rev	GAATCGAAGTGCAAGCATC
f2l-for	TCTAAGATGCTTGCACTTC
f2l-rev	GTGCCTTCGCCATATCAG
fimX-for	GAGGCGTCTAATAATCTTG
fimX-rev	GTCATACATCGGCGTCAG

### Analysis and categorization of fimbrial subunit types

Nucleotide sequences encoding predicted fimbrial subunit genes were extracted from all *fimX* locus nucleotide sequences and identical sequences were identified and grouped into fimbrial subunit gene types using seqkit v.2.4.0 [38]. For each fimbrial subunit gene type consisting of identical nucleotide sequences, a single reference sequence was chosen to represent each fimbrial subunit gene type (S2 Table). Sequence alignment with MAFFT v.7.490 [39] was then used to classify the representative sequences by similarity into related gene types. This analysis resulted in the identification of six fimbrial subunit gene types: *fimbrial protein 4*, *fimbrial protein 3*, *fimbrial protein 1*, *fimN*, *fim2-like*, and *fimX*. Any fimbrial subunit gene sequence that could not be translated into a complete protein sequence, due to a truncation, frameshift, or incomplete sequence, was labeled a pseudogene and excluded from further analysis. In total, 1,145 predicted fimbrial subunit genes were identified, of which 5 were designated as pseudogenes, leaving 1,140 genes used for further analysis. The reference gene sequence from each fimbrial subunit gene type was translated and used to identify similar protein sequences from the unique protein sequences to group all protein sequences into a fimbrial subunit protein type. Eighty-one unique nucleotide sequences encoding predicted fimbrial subunit genes were identified from this analysis, which resulted in sixty-seven unique protein sequences after translation. The recombination detection program 5 (RDP5) was used for detection of recombination events within the 81 unique fimbrial subunit gene sequences using default settings [40]. Only the events that passed two out of seven implemented methods in RDP5 with a significance level (p) < 0.05 were considered true recombination events.

### Phylogenetic analysis

Sequence alignments of both nucleotide and amino acid sequences were performed using MAFFT v.7.490 [39]. Maximum-likelihood phylogenetic trees were inferred using IQ-TREE v.2.4.0 using the automatic model selection process provided by ModelFinder Plus [41,42] and visualized with iTOL v.7.2 [43].

## Results

### *fimX* locus cluster designations

The *fimX* locus was located in the same genomic position flanked by tripartite ATP-independent periplasmic transporter (*TRAP*) and phenylacetate-CoA ligase (*paaK*) genes in all *B. bronchiseptica* genome assemblies analyzed. The diversity within the locus included both the number of genes harbored, as well as the type of predicted fimbrial genes present within the *fimX* locus. These two criteria were used to categorize the locus sequences from all *B. bronchiseptica* genome assemblies into twelve distinct *fimX* locus cluster types. A reference strain sequence was chosen to represent each *fimX* cluster type, which were named cluster type 5 through cluster type 2c (Fig 1 and S1 Table). Poly-cytosine or poly(C) tracts were identified upstream of the annotated start codon for all the predicted fimbrial genes harbored within the *fimX* locus for all the *B. bronchiseptica* genome assemblies evaluated. The metadata associated with all the *B. bronchiseptica* genome assemblies utilized, such as lineage, ST, and host, was evaluated for a correlation between a *fimX* locus cluster type and these attributes. However, no correlation was found (S1Table).

One hundred and forty-six *B. bronchiseptica* genome assemblies were observed to harbor five predicted fimbrial subunit gene types within the *fimX* locus. The five predicted fimbrial subunit gene types harbored by these isolates were *fimbrial protein 3*, *fimbrial protein 1*, *fimN*, *fim2-like*, and *fimX* (Fig 1 and S1 Table). *Fimbrial protein 3* is a recently identified predicted fimbrial subunit gene type [31]. Genome assemblies harboring this configuration of predicted fimbrial gene types were categorized as cluster type 5, which was the most prevalent among all the *B. bronchiseptica* genome assemblies analyzed.

Fifty-six *B. bronchiseptica* genome assemblies harbored four fimbrial subunit gene types that included *fimbrial protein 1, fimN*, *fim2-like*, and *fimX*. Genome assemblies that harbored this configuration of predicted fimbrial subunit gene types were classified into cluster 4a (Fig 1 and S1 Table). Twenty-six *B. bronchiseptica* genome assemblies harbored four fimbrial subunit gene types that included *fimbrial protein 3*, *fimN*, *fim2-like*, and *fimX*. Genome assemblies harboring this configuration of predicted fimbrial gene types were grouped into cluster 4b. Four *B. bronchiseptica* genome assemblies harbored four fimbrial subunit gene types that included *fimbrial protein 3*, *fimbrial protein 1*, *fim2-like*, and *fimX*. These assemblies did not contain a *fimN* gene and were categorized as cluster type 4c.

*B. bronchiseptica* genome assemblies RB50 and FDAARGOS_176 contained three fimbrial subunit gene types that included *fimbrial protein 3*, *fimN*, and *fimX*, and were categorized as cluster type 3a (Fig 1 and S1 Table). Twelve *B. bronchiseptica* genome assemblies harbored three fimbrial subunit gene types that included *fimN*, *fim2-like*, and *fimX*, and were classified into the cluster type 3b. *B. bronchiseptica* genome assemblies D973 and D17-015854 harbored three fimbrial subunit gene types that included *fimbrial protein 3*, *fim2-like*, and *fimX,* and were categorized into cluster type 3c. Both *fimX* locus cluster types 4c and 3c did not contain a *fimN* gene. The genome assembly for strain D16-0377096 harbored three fimbrial subunit gene types that included *fimbrial protein 1*, *fim2-like*, and *fimX,* and was grouped into cluster type 3d. The genome assembly for strain D17-021385 harbored three fimbrial subunit gene types that included *fimbrial protein 1*, *fimN*, and *fimX,* and was placed into cluster type 3e.

Six *B. bronchiseptica* genome assemblies harbored two fimbrial subunit gene types, *fim2-like* and *fimX,* and were classified into the *fimX* locus cluster type 2a (Fig 1 and S1 Table). The genome assembly for strain D16-050946 harbored two fimbrial subunit gene types, *fimbrial protein 4* and *fimX,* and was classified into cluster type 2b. The *fimbrial protein 4* gene is a recently identified predicted fimbrial subunit gene type and the assembly for strain D16-050946 was the only assembly observed to harbor *fimbrial protein 4*. *B. bronchiseptica* genome assemblies F-1 and F2 harbored two fimbrial subunit gene types, *fimbrial protein 1* and *fimX*, and these assemblies were categorized as cluster type 2c.

### Fimbrial subunit types

**Fig 2:**
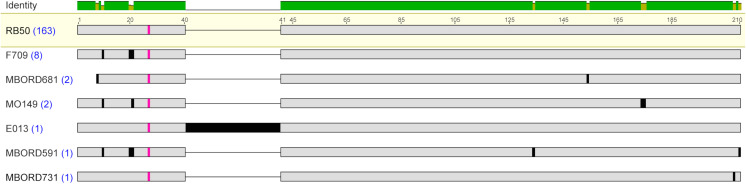
Amino acid sequence alignment of Fimbrial protein 3 sequences. MAFFT alignment of the unique representative Fimbrial protein 3 protein sequences. *B. bronchiseptica* strain shown at left with blue numbers in parentheses indicating the number of genome assemblies found to encode the amino acid sequence. The most prevalent protein sequence was used as the reference in the alignment (top); amino acid residues that differ from the reference are highlighted by black bars and grey indicates amino acid residues that are identical to the reference sequence. The conserved A residue is highlighted in pink. The bar at the top of the alignment represents the mean pairwise identity over all pairs in the column, with green indicating 100% identity and yellow indicating at least 30% and less than 100% identity.

Focusing on each fimbrial subunit gene type, analysis of all the *fimbrial protein 3* genes identified among the *B. bronchiseptica* genome assemblies resulted in the detection of seven unique protein sequences (S2 Table). Fimbrial protein 3 sequences were highly conserved among each other, with a pairwise amino acid identity that ranged from 83.68% to 99.52%, and divergent from the other fimbrial subunit type protein sequences (Table 2). The Fimbrial protein 3 amino acid sequence from strain E013 contained a 35 amino acid internal insertion that was not present in any other Fimbrial protein 3 sequences. Aside from the 35 amino acid insertion, the Fimbrial protein 3 amino acid sequences from strains E013 and RB50 were identical (Fig 2). A conserved Ala residue was observed in all Fimbrial protein 3 amino acid sequences located at residue 20 for MBORD681 and at residue 27 for the other Fimbrial protein 3 amino acid sequences (Fig 2 and Table 5). The majority of the amino acid variations observed among all the Fimbrial protein 3 sequences were located within the N-terminal region, encompassing the start codon to the conserved Ala residue (Fig 2). When the N-terminal region was removed, so that the protein sequence used for comparisons began at the conserved Ala residue and extended to the C-terminal end residue, the upper range of the pairwise amino acid identity increased to 100% (Table 3).

**Table 2:** Pairwise amino acid identity (%) from the full-length protein sequences of fimbrial subunit types.

	Fimbrial protein 3	Fimbrial protein 1	FimN	Fim2-like	FimX	Fimbrial protein 4
Fimbrial protein 3	83.68 - 99.52^a^	58.78 - 82.76	50 - 66.67	49.18 - 67.30	43.85 - 54.41	64.44 - 75.49
Fimbrial protein 1	58.78 - 82.76	81.68 - 99.52	59.05 - 69.05	59.80 - 73.93	52.88 - 59.69	63.05 - 76.85
FimN	50 - 66.67	59.05 - 69.05	77.03 - 99.52	50.24 - 64.76	49.04 - 55.77	70.79 - 82.59
Fim2-like	49.18 - 67.30	59.80 - 73.93	50.24 - 64.76	77.89 - 99.52	51.20 - 75.60	61.08 - 71.43
FimX	43.85 - 54.41	52.88 - 59.69	49.04 - 55.77	51.20 - 75.60	70.53 - 99.50	53.20 - 56.65
Fimbrial protein 4	64.44 - 75.49	63.05 - 76.85	70.79 - 82.59	61.08 - 71.43	53.20 - 56.65	--

^a^Lowest - highest pairwise % identity (excluding self-v-self) from MAFFT multi-sequence alignments of all representative protein sequences (67 sequences)

**Table 3:** Pairwise amino acid identity (%) from the protein sequences of fimbrial subunit types without the N-terminal region.

	Fimbrial protein 3	Fimbrial protein 1	FimN	Fim2-like	FimX	Fimbrial protein 4
Fimbrial protein 3	83.11 – 100^a^	61.64 - 82.07	51.82 - 65.41	51.36 - 64.32	45.91 - 57.30	61.82 - 73.51
Fimbrial protein 1	61.64 - 82.07	86.56 - 100	61.41 - 67.93	62.70 - 70.27	56.52 - 61.96	65.76 - 74.46
FimN	51.82 - 65.41	61.41 - 67.93	83.06 - 100	54.05 - 64.67	53.26 - 60.33	75.41 - 87.91
Fim2-like	51.36 - 64.32	62.70 - 70.27	54.05 - 64.67	84.78 - 99.46	55.14 - 72.43	64.13 - 69.02
FimX	45.91 - 57.30	56.52 - 61.96	53.26 - 60.33	55.14 - 72.43	74.32 - 100	55.43 - 59.24
Fimbrial protein 4	61.82 - 73.51	65.76 - 74.46	75.41 - 87.91	64.13 - 69.02	55.43 - 59.24	--

^a^Lowest - highest pairwise % identity (excluding self-v-self) from MAFFT multi-sequence alignments of all representative protein sequences (67 sequences) trimmed to start at the conserved A residue.

**Table 5:** N-terminal amino acid sequence groups.

N-Terminal Group	Sequence^a^	Length
1	980−2_ Fim2-like	25
	A345_ Fim2-like	
	D17-021385_FimX	
	FDAARGOS_634_ Fim2-like	
	FDAARGOS_693_ Fim2-like	
	KM22_ Fim2-like	
	MBORD707_ Fim2-like	
	MO149_ Fim2-like	
2	A345_FimX	21
	D16-047428_FimX	
	F-1_FimX	
3	I328_FimX	21
	M2020-2_FimX	
4	KM22_FimX	21
	RB50_FimX	
5	FDAARGOS_693_FimX	21
	MBORD707_FimX	
	MO149_FimX	
6	99-R-0433_ Fim2-like	27
	I328_Fimbrial protein 1	
	M2020-2_ Fim2-like	
7	C2020-8_Fimbrial protein 1	27
	MBORD635_FimN	
	RB50_FimN	
8	D15-055809_Fimbrial protein 1	27
	D16-049392_Fimbrial protein 1	
	D755_Fimbrial protein 1	
9	CA90BB1334_FimN	27
	I943_FimN	
	MO149_FimN	
	MO275_FimN	
10	B20-10725633_FimN	27
	D755_ Fim2-like	
	FMDBb1_FimN	
	KM22_FimN	
11	59324_Fimbrial protein 1	27
	F709_Fimbrial protein 3	
	MBORD591_Fimbrial protein 3	
12	D16-047428_FimN	20
	F-1_Fimbrial protein 1	
13	59327_Fimbrial protein 1	20
	A345_FimN	
14	E013_Fimbrial protein 3	27
	MBORD731_Fimbrial protein 3	
	RB50_Fimbrial protein 3	
	RL57_ Fim2-like	
15	253_Fimbrial protein 1	20
	B20-10725633_Fimbrial protein 1	
	D16-011513_Fimbrial protein 1	
	D16-014064_Fimbrial protein 1	
	D16-20785_Fimbrial protein 1	
	D16-041144_Fimbrial protein 1	
	D16-050946_Fimbrial protein 4	
	D17-007548_FimN	
	KM22_Fimbrial protein 1	
	MBORD678_Fimbrial protein 1	
	MBORD681_Fimbrial protein 3	
Unique	MO149_Fimbrial protein 3	27
	FDAARGOS_693_Fimbrial protein 1	20
	FDAARGOS_542_Fimbrial protein 1	13
	MBORD707_Fimbrial protein 1	27
	I328_FimN	27
	F709_FimN	27
	D16-046502_FimN	13
	MBORD707_FimN	27
	I328_ Fim2-like	25
	D445_FimX	21

^a^Sequence listed as strain name_fimbrial subunit type

Examination of the proteins encoded by the *fimbrial protein 1* genes identified among the *B. bronchiseptica* genome assemblies resulted in the identification of nineteen unique protein sequences (S2 Table). The Fimbrial protein 1 amino acid sequences were highly conserved among each other, with a pairwise amino acid percent identity that ranged from 81.68% to 99.52%, and divergent from the other fimbrial subunit type protein sequences (Table 2). All the unique Fimbrial protein 1 amino acid sequences were from strains belonging to *B. bronchiseptica* lineage I, except for strain I328, which is a *B. bronchiseptica* lineage II strain (S1 Table). The lowest pairwise amino acid percent identity was observed for the Fimbrial protein 1 sequence from strain I328 (S3 Table). This is consistent with previous reports demonstrating that virulence factors and vaccine antigens encoded by genes from *B. bronchiseptica* lineage II strains have a lower sequence similarity to factors encoded by paralogous genes from *B. bronchiseptica* lineage I strains [32,33]. When the Fimbrial protein 1 sequence from strain I328 was excluded, the pairwise amino acid identity range increased to 84.62% to 99.52% (Table 4).

**Table 4:** Pairwise amino acid identity (%) from the full-length protein sequences of fimbrial subunit types from Lineage I strains.

	Fimbrial protein 3	Fimbrial protein 1	FimN	Fim2-like	FimX	Fimbrial protein 4
Fimbrial protein 3	83.68 - 99.52^a^	58.78 - 82.76	50 - 68.14	49.18 - 67.30	44.67 - 54.41	64.44 - 75.49
Fimbrial protein 1	58.78 - 82.76	84.62 - 99.52	59.61 - 69.05	60.29 - 69.12	52.40 - 59.18	63.05 - 76.85
FimN	50 - 68.14	59.61 - 69.05	80.38 - 99.52	51.20 - 61.61	51.44 - 55.17	73.76 - 82.59
Fim2-like	49.18 - 67.30	60.29 - 69.12	51.20 - 61.61	88.94 - 99.52	55.29 - 76.44	63.55 - 71.43
FimX	44.67 - 54.41	52.40 - 59.18	51.44 - 55.17	55.29 - 76.44	79.71 - 99.50	56.93 - 57.43
Fimbrial protein 4	64.44 - 75.49	63.05 - 76.85	73.76 - 82.59	63.55 - 71.43	56.93 - 57.43	--

^a^Lowest - highest pairwise % identity (excluding self-v-self) from MAFFT multi-sequence alignments of all representative protein sequences from Lineage I strains only (60 sequences).

**Fig 3:**
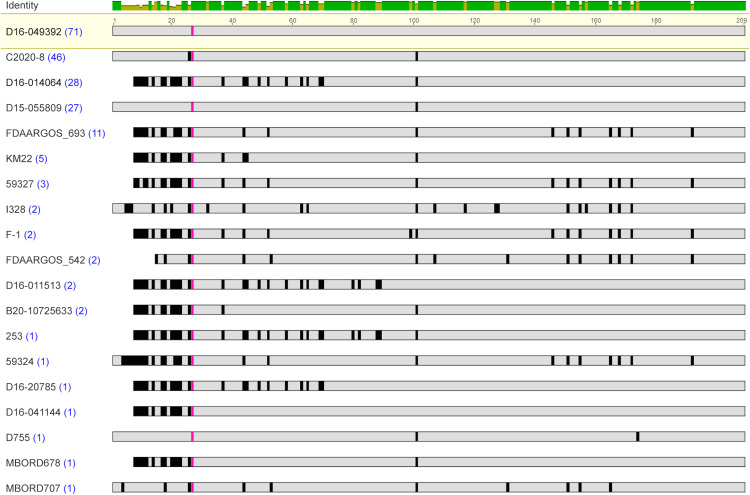
Amino acid sequence alignment of Fimbrial protein 1 sequences. MAFFT alignment of the unique representative Fimbrial protein 1 protein sequences. *B. bronchiseptica* strain shown at left with blue numbers in parentheses indicating the number of genome assemblies found to encode the amino acid sequence. The most prevalent protein sequence was used as the reference in the alignment (top); amino acid residues that differ from the reference are highlighted by black bars and grey indicates amino acid residues that are identical to the reference sequence. The conserved A residue is highlighted in pink. The bar at the top of the alignment represents the mean pairwise identity over all pairs in the column, with green indicating 100% identity and yellow indicating at least 30% and less than 100% identity.

Similar to the Fimbrial protein 3 sequences, a conserved Ala residue was observed in all Fimbrial protein 1 sequences located at residues 13–27 (Table 5). The majority of the amino acid variations observed among all the Fimbrial protein 1 sequences were also located within the N-terminal region, encompassing the start codon up to the residue preceding the conserved Ala residue (Fig 3). When the N-terminal region was removed, so that the protein sequence used for comparisons began at the conserved Ala residue and extended to the C-terminal end residue, the amino acid identity range increased to 86.89% to 100% (Table 3).

Assessment of the proteins encoded by the *fimN* genes identified among the *B. bronchiseptica* genome assemblies resulted in the detection of sixteen unique protein sequences (S2 Table 2). The FimN protein sequences were highly conserved among each other, with a pairwise amino acid identity that ranged from 77.03% to 99.52%, and divergent from the other fimbrial subunit type protein sequences (Table 2). Similar to the Fimbrial protein 1 sequence, the lowest pairwise amino acid percent identity was observed for the FimN sequence from strain I328 and when the FimN sequence from strain I328 is excluded, the amino acid identity range increased to 80.38% to 99.52% (Table 4). A low pairwise amino acid percent identity was also observed for the FimN protein sequence from strain RB50, with a pairwise amino acid percent identity that ranged from 86.89% to 90.70%. The amino acid differences in FimN from RB50 were mainly located within a 40-residue region located at the C-terminus.

**Fig 4:**
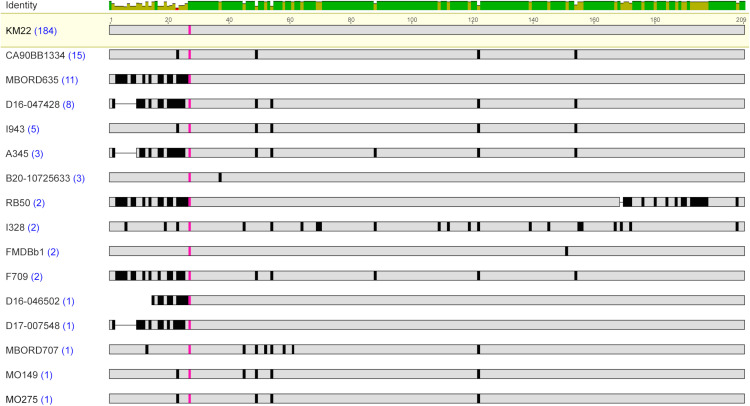
Amino acid sequence alignment of FimN sequences. MAFFT alignment of the unique representative FimN protein sequences. *B. bronchiseptica* strain shown at left with blue numbers in parentheses indicating the number of genome assemblies found to encode the amino acid sequence. The most prevalent protein sequence was used as the reference in the alignment (top); amino acid residues that differ from the reference are highlighted by black bars and grey indicates amino acid residues that are identical to the reference sequence. The conserved A residue is highlighted in pink. The bar at the top of the alignment represents the mean pairwise identity over all pairs in the column, with green indicating 100% identity, yellow indicating at least 30% and less than 100% identity, and red indicating less than 30% identity.

Similar to the other fimbrial subunit type protein sequences, a conserved Ala residue was observed in all FimN sequences at residue 16 for FimN from strain D16-046502 and at residue 27 for the other FimN sequences (Table 5). The majority of the amino acid variations observed among all the FimN sequences were located within the N-terminal region, encompassing the start codon to the conserved Ala residue (Fig 4). When the N-terminal region was removed, so that the protein sequence used for comparisons began at the conserved Ala residue and extended to the C-terminal end residue, the amino acid identity range increased to 83.06% to 100% (Table 3).

Investigation of the *fim2-like* genes identified among the *B. bronchiseptica* genome assemblies resulted in the identification of twelve unique protein sequences (S2 Table). Fim2-like protein sequences were highly conserved among each other, with a pairwise amino acid identity that ranged from 77.89% to 99.52%, and divergent from the other fimbrial subunit type protein sequences (Table 2). The lowest pairwise amino acid percent identity was observed the Fim2-like protein sequence from strains M2020-2, I328, and 99-R-0433, which belong to Bb lineage II. When these more diverse Fim2-like protein sequences from Bb lineage II isolates were excluded, the amino acid identity range increased to 88.94% to 99.52% (Table 4).

**Fig 5:**
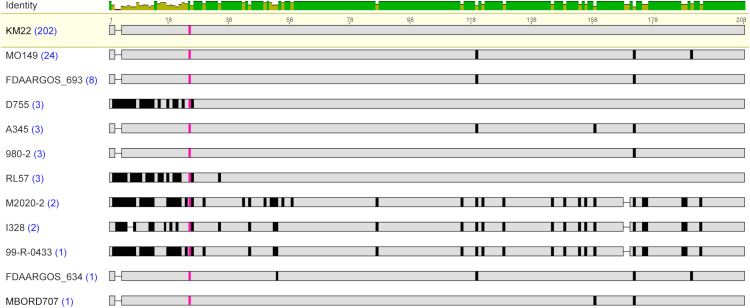
Amino acid sequence alignment of Fim2-like sequences. MAFFT alignment of the unique representative Fim2-like protein sequences. *B. bronchiseptica* strain shown at left with blue numbers in parentheses indicating the number of genome assemblies found to encode the amino acid sequence. The most prevalent protein sequence was used as the reference in the alignment (top); amino acid residues that differ from the reference are highlighted by black bars and grey indicates amino acid residues that are identical to the reference sequence. The conserved A residue is highlighted in pink. The bar at the top of the alignment represents the mean pairwise identity over all pairs in the column, with green indicating 100% identity, yellow indicating at least 30% and less than 100% identity, and red indicating less than 30% identity.

Similar to the protein sequences from the other fimbrial subunit type protein sequences, a conserved Ala residue was observed in all Fim2-like protein sequences at residue 27 for Fim2-like from strains 99-R-0433, M2020-2, D755, and RL57 and at residue 25 for the other Fim2-like protein sequences (Fig 5 and Table 5). The majority of the amino acid variations observed among all the Fim2-like protein sequences were located within the N-terminal region, encompassing the start codon to the conserved Ala residue (Fig 5). When the N-terminal region was removed, so that the protein sequence used for comparisons began at the conserved Ala residue and extended to the C-terminal end residue, the amino acid identity range increased to 84.78% to 99.46% (Table 3).

Analysis of the *fimX* genes identified among the *B. bronchiseptica* genome assemblies resulted in the detection of twelve unique protein sequences (S2 Table). FimX protein sequences were highly conserved among each other, with a pairwise amino acid identity that ranged from 70.53% to 99.50%, and divergent from the other fimbrial subunit type protein sequences (Table 2). The lowest pairwise amino acid percent identity was observed for the FimX protein sequence from strains M2020-2 and I328, which belong to Bb lineage II. When these FimX protein sequences were excluded, the amino acid identity range increased to 79.71% to 99.50% (Table 4).

**Fig 6:**
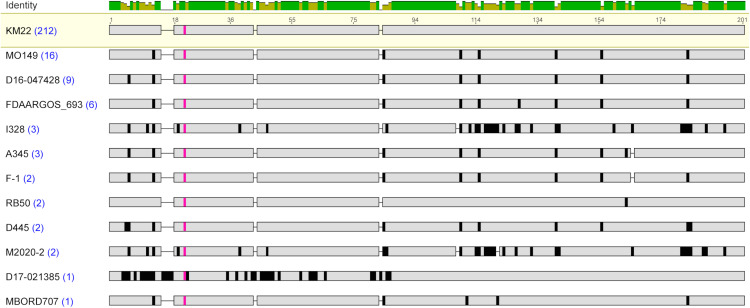
Amino acid sequence alignment of FimX sequences. MAFFT alignment of the unique representative FimX protein sequences. *B. bronchiseptica* strain shown at left with blue numbers in parentheses indicating the number of genome assemblies found to encode the amino acid sequence. The most prevalent protein sequence was used as the reference in the alignment (top); amino acid residues that differ from the reference are highlighted by black bars and grey indicates amino acid residues that are identical to the reference sequence. The conserved A residue is highlighted in pink. The conserved A residue is highlighted in pink. The bar at the top of the alignment represents the mean pairwise identity over all pairs in the column, with green indicating 100% identity and yellow indicating at least 30% and less than 100% identity.

A conserved Ala residue was observed in all FimX protein sequences at residue 25 for FimX from strain D17-021385 and at residue 21 for the other Fim2-like protein sequences (Fig 6 and Table 5). The majority of the amino acid variations observed among all the Fim2-like protein sequences were located within the N-terminal region, encompassing the start codon to the conserved Ala residue (Fig 6). When the N-terminal region was removed, so that the protein sequence used for comparisons began at the conserved Ala residue and extended to the C-terminal end residue, the amino acid identity range increased to 74.32% to 100% (Table 3).

**Fig 7:**
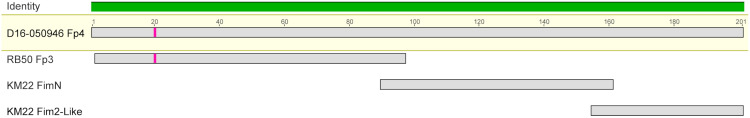
Fimbrial protein 4 alignment. MAFFT alignment of Fimbrial protein 4 protein sequence with regions of RB50 Fimbrial protein 3, KM22 FimN, and KM22 Fim2-like are 100% identical. Amino acid residues 9 - 104 of RB50 Fimbrial protein 3 are 100% identical to amino acid residues 2 - 97 of Fimbrial protein 4. Amino acid residues 97 - 168 of KM22 FimN are 100% identical to amino acid residues 90 - 161 of Fimbrial protein 4. Amino acid residues 162 - 208 of KM22 Fim2-like are 100% identical to amino acid residues 155 - 201 of Fimbrial protein 4. The conserved A residue is highlighted in pink. The bar at the top of the alignment represents the mean pairwise identity over all pairs in the column, with green indicating 100% identity.

The *fimbrial protein 4* gene found in strain D16-050946 was the only *fimbrial protein 4* gene found among the *B. bronchiseptica* genome assemblies evaluated. It was recently identified as a new predicted fimbrial subunit gene within the *fimX* locus due to its low nucleotide sequence identity to the other known predicted fimbrial subunit genes [31]. Pairwise comparisons of both the full-length and the trimmed portion in which the N-terminal region was removed, such that the protein sequences used for comparisons began at the conserved Ala residue and extended through the C-terminal end residue, of Fimbrial protein 4 sequence to the other fimbrial subunit type protein sequences demonstrated an overall low amino acid identity to other fimbrial subunit type protein sequences (Table 2 and Table 3). Further examination of the Fimbrial protein 4 amino acid sequence to other fimbrial subunit type protein sequences revealed that the Fimbrial protein 4 sequence shared regions with 100 percent amino acid identity with regions from three other fimbrial subunit type protein sequences. Specifically, residues 2–97 of Fimbrial protein 4 were 100% identical to 9–104 of RB50 Fimbrial protein 3, residues 90–161 of Fimbrial protein 4 were 100% identical to residues 97–168 of KM22 FimN, and residues 155–201 of Fimbrial protein 4 were 100% identical to162–208 of KM22 Fim2-like (Fig 7). Additionally, the N-terminal region encompassing, residues 1–20 of Fimbrial protein 4 were 100% identical to the N-terminal regions of the following fimbrial subunit types: MBORD681 Fimbrial protein 3, D17-007548 FimN, and Fimbrial protein 1 from strains D16-011513, D16-20785, B20-10725633, D16-041144, D16-014064, 253, MBORD678, and KM22. This data suggests that new fimbrial subunit genes are likely generated by homologous recombination between existing or ancestral fimbrial subunit genes located within the *fimX* locus.

### N-terminal region

To further evaluate the N-terminal region, the N-terminal amino acid sequences from all fimbrial subunit types were analyzed and placed into groups based on amino acid identity. This resulted in 15 N-terminal groups each consisting of two or more identical amino acid sequences and one group comprised of 10 unique N-terminal amino acid sequences (Table 5). The majority of the N-terminal sequence groups contained sequences from different fimbrial subunit types despite sharing 100% identity. For example, the N-terminal amino acid sequence of FimX from strain D17-021385 was 100% identical to the N-terminal amino acid sequence of Fim2-like from strains 980−2, A345, FDAARGOS_634, FDAARGOS_693, KM22, MBORD707, and MO149 (S5 Table and Table 5). The N-terminal amino acid sequence from all of these strains comprised N-terminal group 1 (Table 5).

**Fig 8:**
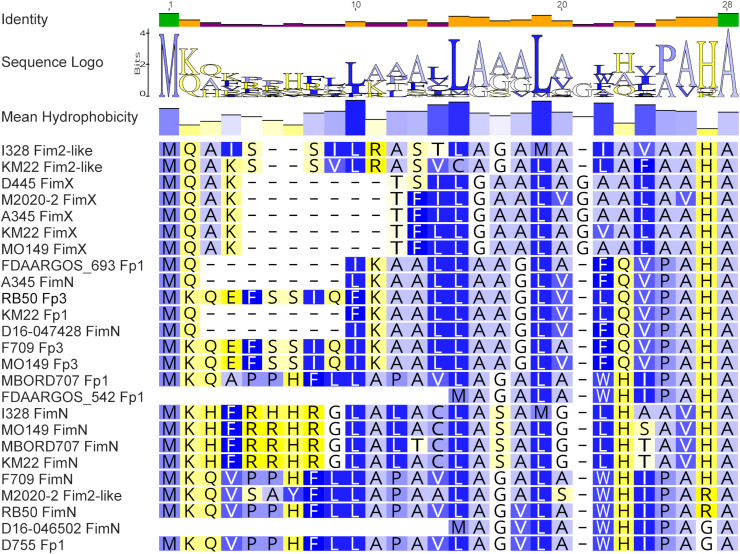
N-terminal amino acid sequence alignment. Alignment includes a single representative N-terminal sequence from each N-terminal group designation [12–15] and all unique N-terminal sequences listed in Table 5. Residues are colored by hydrophobicity values [59] in a graduated color scheme from strongly hydrophobic (dark blue) to strongly hydrophilic (bright yellow), with glycine as the midpoint in white. B. bronchiseptica strain shown at left with fimbrial subunit type name. Fp1 is used to indicate Fimbrial protein 1 and Fp3 is used to indicate Fimbrial protein 3. The bar at the top of the alignment represents the mean pairwise identity over all pairs in the column, with green indicating 100% identity, yellow at least 30% and less than 100% identity, and red less than 30% identity.

Next, a representative amino acid sequence was chosen from each N-terminal amino acid sequence group and aligned along with each of the ten unique N-terminal amino acid sequences revealing several conserved sequence motifs. An overall pattern of hydrophobic residues followed by short stretches of several hydrophilic residues or neutral residues were observed throughout the N-terminal sequence alignment (Fig 8). Additionally, a conserved Leu at residues 15 and 19 were detected and observed to be part of a conserved sequence motif of Leu, Ala, Ala, Ala, Leu, and Ala at residues 15–20. Another conserved sequence motif consisted of Met, Lys, and Gln at residues 1–3 and a Pro, Ala, His, and Ala conserved sequence motif at residues 25–30 was additionally observed.

### Phylogenic and recombination analysis

**Fig 9:**
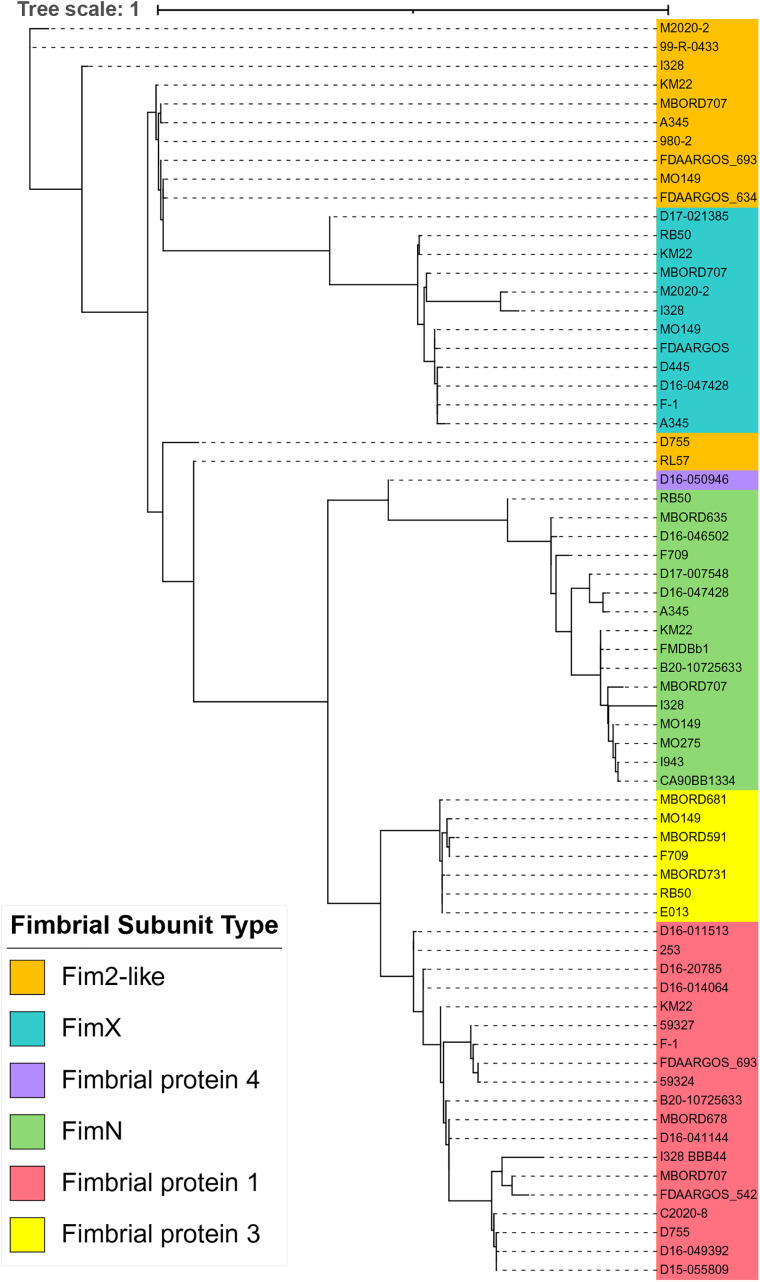
Maximum-likelihood phylogenetic tree inferred from the full-length protein sequences of fimbrial subunit types. *B. bronchiseptica* strain names colored according to the legend shown at the left of tree.

A phylogenetic tree inferred from the alignment of all the full-length representative fimbrial subunit type protein sequences resulted in an overall tree topology in which all fimbrial subunit

protein sequences from each fimbrial subunit type clustered together (Fig 9). Fim2-like protein sequences were the exception to the overall tree topology. The Fim2-like proteins shared a common ancestor with the remainder of the subunit protein types, but were polyphyletic, with some singletons more closely related to different proteins.

**Fig 10:**
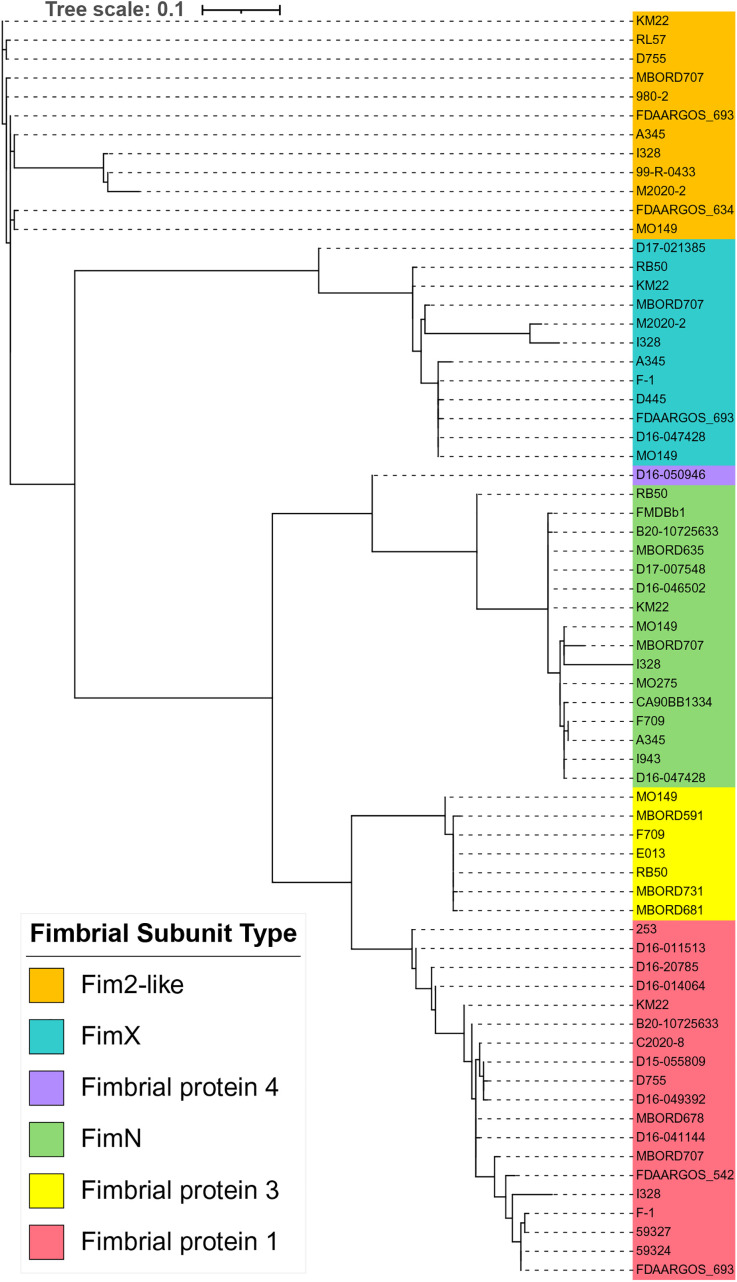
Maximum-likelihood phylogenetic tree inferred from the protein sequences of fimbrial subunit types without the N-terminal region. The N-terminal region was removed for all protein sequences, such that the protein sequences used for comparisons began at the conserved A residue and extended through the C-terminal end residue.

When the N-terminal region was removed from all representative fimbrial subunit type protein sequences and the more conserved region of the proteins that begins at the conserved Ala residue and extends to the C-terminal end residue was used to infer the phylogenetic relationships, the resulting tree topology extensively aligned with the distribution of fimbrial gene types (Fig 10). Notably, the Fim2-like protein sequences of strains D755 and RL57 were placed in the same cluster as other Fim2-like protein sequences, demonstrating that the separation of these protein sequences from other Fim2-like protein sequences, when whole protein sequences were compared, was likely due to the N-terminal region (Fig 9).

**Fig 11:**
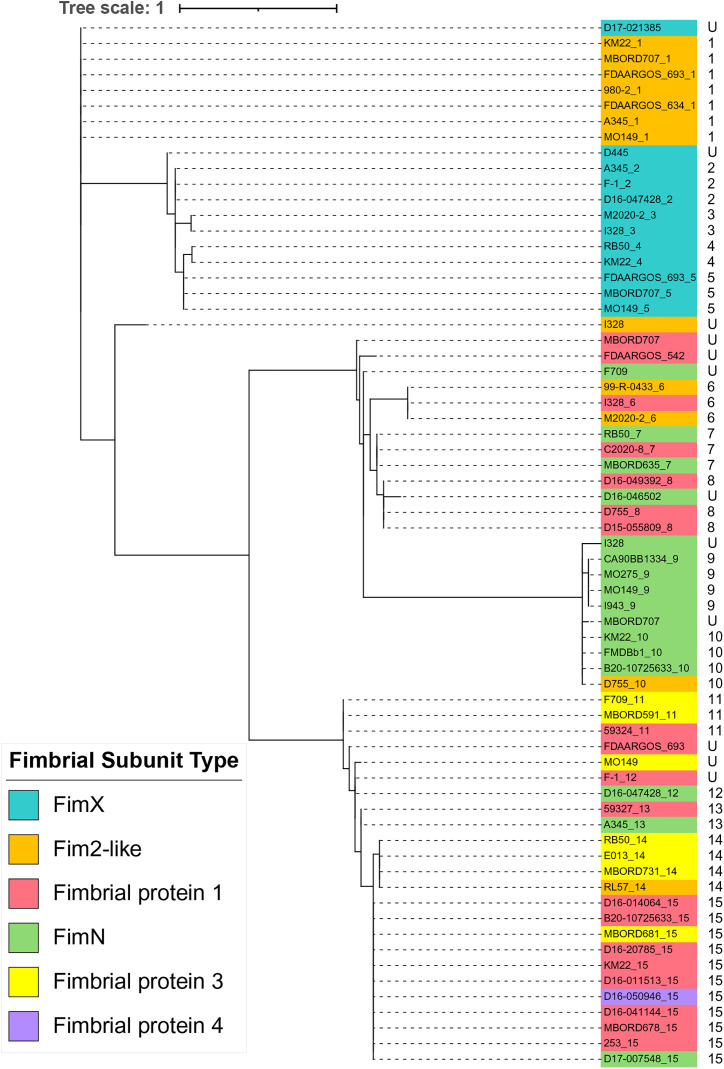
Maximum-likelihood phylogenetic tree inferred from the N-terminal amino acid sequences of fimbrial subunit types. The N-terminal region used included the start codon up to and including the conserved A residue. *B. bronchiseptica* strain names colored according to the legend shown at the left of tree. N-terminal group designations, 1-15 and unique indicated by U, provided alongside strain names on the right side.

When the N-terminal regions, beginning at the start codon up to and including the conserved Ala residue, from all representative fimbrial subunit type protein sequences were used to infer a phylogeny, the resulting tree topology did not align with the distribution of fimbrial gene types (Fig 11). In fact, a high degree of variation was observed with the N-terminal sequences from the fimbrial subunit types interspersed throughout the tree. The exception to this distribution was the FimX and FimN N-terminal sequences, in which most of the sequences grouped together based on fimbrial subunit type (Fig 11). However, while the tree topology did not align with the distribution of fimbrial gene types, the N-terminal sequences did group together based on N-terminal group designations. This resulting distribution was likely, given that the N-terminal group designations are based on amino acid identity. Collectively, the detailed phylogenic inferences suggest that diversity among the fimbrial subunit genes located within the *fimX* locus was likely the result of recombination events.

To further explore the likelihood that genetic diversity within the *fimX* locus was driven by local recombination events, all unique fimbrial subunit gene sequences were evaluated for local recombination events. Fifteen separate recombination events were detected using at least two statistics-based methods (S6 Table). The recombinant genes were the result of recombination between parent genes from different gene families. For example, recombination between a *fimN* gene and a *fim2-like* gene resulted in a new *fimN* gene sequence. The majority of the detected recombination events were located at the 5-prime end of the gene sequences, consistent with the observation that the phylogenetic tree inferred from N-terminal sequences was topologically distinct from the phylogenetic tree inferred from the fimbrial protein sequences with the N-terminus removed (Fig 10 and Fig 11). Two distinct recombination events were detected in the fimbrial protein 4 gene found in strain D16-050946, consistent with the observation that the protein sequence contains regions similar to three different fimbrial proteins (S6 Table and Fig 7). Combined, the data from phylogenic and recombination analysis suggests that diversity among the fimbrial subunit genes located within the *fimX* locus was likely the result of homologous recombination.

## Discussion

Comparative genomic analysis of *B. bronchiseptica* strains to date have shown limited genomic variability [8,9,31–33,44]. One of the few regions in which genomic variability has been reported is within the *fimX* locus. In this report, we analyzed 259 *B. bronchiseptica* genome assemblies to evaluate the genetic variability located within the *fimX* locus. Despite the sequence diversity within the *fimX* locus, the locus was located in the same genomic location flanked by the TRAP transporter and *paaK* genes in all *B. bronchiseptica* genome assemblies analyzed. The genomic organization of the *fimX* locus in *B. bronchiseptica* differs from the genomic organization of the *fimX* locus in *B. pertussis* strains, in which the locus only contains the *fimX* gene and the ends of the locus are chromosomally separated. In *B. pertussis* assemblies, a transposase gene is co-located next to the 3-prime end of the *TRAP* gene with approximately 60 kb or more sequence, depending on the *B. pertussis* assembly, separating those genes from another transposase gene co-located next to *fimX*, followed by the *paaK* gene. This difference in gene order is likely the result of genomic rearrangements induced by insertion sequence elements harbored by *B. pertussis* isolates [45,46]. In fact, while *B. bronchiseptica* genomes rarely contain any insertion sequence elements, genomes of *B. pertussis* isolates contain numerous insertion sequence elements, which have resulted in genome reduction and genome rearrangements [5,9,45–47].

The genetic variability identified within the *fimX* locus included both the number of genes harbored, as well as the type of predicted fimbrial subunit genes within the locus. A total of six different fimbrial subunit gene types were identified among the *B. bronchiseptica* genome assemblies analyzed in this study. Poly(C) tracts were identified upstream of all the predicted fimbrial gene types harbored within the *fimX* locus for all the *B. bronchiseptica* genome assemblies evaluated, suggesting that they are regulated by phase-variation, a reversible switching ON or OFF of gene expression [48,49]. Different configurations consisting of the type of predicted fimbrial genes harbored and the number of predicted fimbrial genes present were used to categorize the locus sequence from all *B. bronchiseptica* genome assemblies into twelve distinct *fimX* locus cluster types.

Six different fimbrial subunit gene types were identified among the *B. bronchiseptica* genome assemblies evaluated in this study. The protein sequences, encoded from each specific fimbrial subunit gene type, were highly conserved among each other and divergent from the other protein sequences encoded by the different fimbrial subunit gene types located within the *fimX* locus. This was demonstrated in the phylogenetic tree inferred from the alignment of all the full-length representative fimbrial subunit type protein sequences, which resulted in an overall tree topology in which protein sequences from each fimbrial subunit type tended to form monophyletic groups. The protein sequences encoded from any specific fimbrial subunit gene type from strains belonging to *B. bronchiseptica* lineage II strains had a lower sequence similarity to paralogous genes from *B. bronchiseptica* lineage I strains. This is consistent with previous studies documenting that proteins encoded by genes from *B. bronchiseptica* lineage II strains have a lower sequence similarity to factors encoded by paralogous genes from *B. bronchiseptica* lineage I strains [32,33]. A conserved Ala residue located toward the N-terminal region was identified in every fimbrial protein sequence analyzed. Most of the amino acid variations observed among all the fimbrial protein sequences were located between the start codon and the conserved Ala residue, which was subsequently referred to as the N-terminal region. For all fimbrial protein sequences, the region starting from the Ala residue and extending to the C-terminal end residue was more conserved.

When the N-terminal amino acid sequences from all fimbrial subunit types were analyzed, 15 N-terminal groups consisting of at least two identical amino acid sequences and one group comprised of 10 unique N-terminal amino acid sequences were identified. The majority of the N-terminal sequence groups contained amino acid sequences from different fimbrial subunit types despite sharing 100% identity. This was similarly reflected in the tree topology observed from the phylogenetic analysis using the N-terminal sequences. Specifically, the tree topology did not align with the distribution of fimbrial gene types, but instead the N-terminal sequences clustered together based on N-terminal group designations.

The *Bordetella* fimbriae are members of the type I pili family and are assembled and exported by the chaperone–usher pathway, which has been extensively investigated in UPEC [24–30]. UPEC pilus subunits have a highly conserved N-terminal extension containing a conserved motif of alternating hydrophobic residues, which serve a key role in the donor strand exchange mechanism during pilus assembly. While the precise alternating hydrophobic residues categorized for UPEC pilus subunits were not found in the *B. bronchiseptica* N-terminal sequences, a pattern of hydrophobic residues followed by short stretches of several hydrophilic residues or neutral residues was found, along with two other conserved sequence motifs. These conserved motifs located within the N-terminal region of all fimbrial subunit type protein sequences suggest a functional role in the assembly and export pathway.

Data from both phylogenic and recombination analyses suggest that the diversity within the *fimX* locus was likely driven by recombination. For instance, data showing that the majority of the detected recombination events were located at the 5-prime end of the gene sequences supports the tree topology resulting from the N-terminal sequences, in which the N-terminal sequences grouped together based on N-terminal group designations instead of aligning with the distribution of fimbrial gene types. Additionally, two distinct recombination events were detected in the *fimbrial protein 4* gene, consistent with the Fimbrial protein 4 amino acid sequence containg regions with 100 percent identity with regions from Fimbrial protein 3, FimN, and Fim2-like protein sequences. Similar observations have been documented previously for the *Staphylococcus aureus* enterotoxin genes located within the enterotoxin gene cluster (*egc*), in which new enterotoxin genes have been generated by recombination [50].

*B. pertussis* isolates are known to produce two fimbrial serotypes, referred to as Fim2 and Fim3 [51]. Both are included as components of current acellular pertussis vaccines [52] and several studies have advocated for their inclusion in new vaccine designs [53–58]. Due to the medical importance of whooping cough, the vast majority of studies investigating the expression of fimbrial subunit genes and subsequent production of fimbrial serotypes have focused on *B. pertussis* isolates. Given that *B. bronchiseptica* genomes contain more fimbrial subunit genes than genomes of *B. pertussis* isolates, studies investigating which fimbrial serotypes *B. bronchiseptica* isolates produce are needed. The functional role of *B. bronchiseptica* fimbriae in adherence to respiratory epithelium and any potential role as a protective antigen has come from studies utilizing a *B. bronchiseptica* strain that is incapable of producing any fimbrial serotypes due to an in-frame deletion mutation of the *fimBCD* genes. [35,55]. Vaccines targeting UPEC fimbrial subunits and therapies targeting the blocking of binding and subsequent function of UPEC fimbrial subunits have been successful and are currently undergoing human clinical trials [17–21]. Focusing on veterinary applications, variations of a vaccine targeting multiple fimbrial subunits harbored by enterotoxigenic *Escherichia coli* was demonstrated to be broadly protective against porcine post‑weaning diarrhea [22,23]. Given the success of therapies and vaccines targeting fimbrial antigens in both human and veterinary applications, future efforts targeting the fimbrial antigens harbored by *B. bronchiseptica* offer a promising avenue for veterinary therapies needed to decrease the *B. bronchiseptica* disease burden among animals.

## Supporting information

S1 Table Dataset information. The table lists all strains, RefSeq accession numbers, and strain-associated metadata.
(XLSX)

S2 Table Representative nucleotide and protein sequence information. The table lists the representative nucleotide and protein sequences in the format “strain name: locus_tag number: fimbrial subunit type name”; “fp3” = fimbrial protein 3, “fp1” = fimbrial protein 1, “fp4” = fimbrial protein 4, “UG” = unique group.
(XLSX)

S3 Table Pairwise amino acid identity (%) for all full-length protein sequences of fimbrial subunit types. The table lists the pairwise amino acid identity (%) from MAFFT multi-sequence alignments of all representative full-length protein sequences of fimbrial subunit types.
(XLSX)

S4 Table Pairwise amino acid identity (%) for all protein sequences of fimbrial subunit types without the N-terminal region. The table lists the pairwise amino acid identity (%) from MAFFT multi-sequence alignments of all representative full-length protein sequences in which the N-terminal region was removed, such that the protein sequences used for comparisons began at the conserved A residue and extended through the C-terminal end residue.
(XLSX)

S5 Table Pairwise amino acid identity (%) for all N-terminal amino acid sequences of fimbrial subunit types. The table lists the pairwise amino acid identity (%) from MAFFT multi-sequence alignments of all N-terminal region sequences that include the start codon up to residue preceding the conserved A residue.
(XLSX)

S6 Table Detected recombination events. The table lists the recombination events detected within the 81 unique fimbrial subunit gene sequences using RDP5 [40]. Fimbrial subunit genes with a recombination event (Recombinant) are listed along with the contributing genes (Major Parent, Minor Parent). The representative nucleotide and protein sequence names, and N-terminal region sequence group (see Supplemental Table 2, Table 5) are listed for each recombination event. The “Found In” column lists the number of times the identified recombination event occurred in the gene sequences analyzed. The “+” in the “Detection Methods” column indicates the evaluation method that detected the recombination event (R = RDP, G = GENECONV, B = BOOTSCAN, M = MAXCHI, C = CHIMERA, S = SISCAN, T = 3SEQ).
(XLSX)
